# In Preparation for Outdoor Pharming: Griffithsin Can Be Expressed in *Nicotiana excelsiana* and Retains Activity After Storage as Silage

**DOI:** 10.3389/fbioe.2020.00199

**Published:** 2020-03-18

**Authors:** Paul Eapen, Jennifer Cates, Rich Mundell, Kenneth E. Palmer, Joshua L. Fuqua

**Affiliations:** ^1^University of Louisville School of Medicine, University of Louisville, Louisville, KY, United States; ^2^University of Kentucky College of Agriculture, Food and Environment, University of Kentucky, Lexington, KY, United States; ^3^Department of Pharmacology and Toxicology, James Graham Brown Cancer Center, Center for Predictive Medicine, University of Louisville School of Medicine, University of Louisville, Louisville, KY, United States

**Keywords:** griffithsin, microbicide, antiviral, silage, ensile, *N. excelsiana*

## Abstract

Griffithsin is an algae-derived lectin with strong anti-viral activity against HIV and a positive safety profile. Multiple clinical studies are investigating griffithsin's utility as topical HIV microbicide. HIV microbicides are an extremely cost-sensitive market and plant-based griffithsin protein expression has the potential to meet those demands. The griffithsin product used in the clinic has been expressed and purified in *N. benthamiana*, using a TMV-based viral vector system, Geneware®. Outdoor pharming of biopharmaceuticals would further alleviate startup costs for biotechnology firms and may allow broader product accessibility. Therefore, this study assessed expression in a hybrid tobacco line, *N. excelsiana*, that is susceptible to TMV-based viral vectors and can be grown outdoors. In addition to using this hybrid line we expand on methods for *in planta* storage of griffithsin in leafy plants by ensiling kilogram quantities of griffithsin. The ensiling process allows year-round biomanufacturing, minimal environmental-controlled storage, and reduces the industry need for multiple growth areas to maintain multi-product manufacturing of plant-based pharmaceuticals. This study shows that griffithsin can be expressed in *N. excelsiana* and is stable, recoverable, and active from ensiled tissue. These studies can pave the way for future plant-based pharmaceuticals to be expressed and stored in this manner.

## Introduction

Human Immunodeficiency Virus (HIV) is one of many diverse enveloped viruses that can cause life-threatening human disease. As the global HIV infected population continues to grow, there has become an urgent need to find new microbicides that can block viral entry into human cells (Lusvarghi and Bewley, 2016). While there have been many potential new therapies to treat HIV, significant research has been done on microbicides that can be topically applied to combat HIV. Pre-exposure prophylaxis has been shown to be one of the most effective ways to prevent HIV transmission; however, various issues, such as cost, side effects, access, and adherence have led to less than ideal effectiveness of these drugs (Abdool Karim et al., 2010; Marrazzo et al., 2015; Vamvaka et al., 2016; Marrazzo, 2017). This is particularly evident in rural and developing regions, where access to these drugs may be difficult or non-existent. HIV is particularly devastating in sub-Saharan Africa where it accounts for more than 70% of all global cases (Kharsany and Karim, 2016). In this region, one in 20 people is HIV^+^ and up to 8 million people do not have access to basic medications to treat HIV. Therefore, there is a particular need to develop a high-quality stable virucide that is able to be transported to diverse regions in less-than-ideal environmental conditions that would retain activity to combat the virus months or years after it has been manufactured.

Griffithsin (GRFT) is a lectin derived from the red alga *Griffithsia* sp. that has been shown to exhibit broad-spectrum antiviral activity against HIV in picomolar concentrations (Mori et al., 2005; Emau et al., 2007; Vamvaka et al., 2016). GRFT's antimicrobial activity comes from its ability to bind to viral envelope glycoproteins, specifically to terminal mannoses on oligosaccharides that are present in several enveloped viruses, such as HIV-1, HCV, and SARS-CoV (O'Keefe et al., 2010; Barton et al., 2014, 2016). This binding activity to surface glycoproteins blocks the virus from entering into human cells. In the case of HIV, GRFT binds to the surface glycoprotein gp120 and blocks the virus from binding to its receptors on host cells (Hoelscher et al., 2018). GRFT has shown to be a very effective anti-viral against HIV, and a previous study has shown that manufacturing GRFT at an industrial scale is feasible and easily scalable (Fuqua et al., 2015a,b). In addition, the manufacturing process was assessed and found to have a highly favorable environmental output index while still having almost no risk to health and safety (Alam et al., 2018).

To develop GRFT as an affordable antiviral, mass production at a commercial scale is required for feasibility and low costs to the consumer. This production would ideally be from an easy-to-use construct that is easily scalable and able to be stored and transported with minimal cold chain requirements. GRFT can only be isolated in small quantities from the native algae species, so various studies have looked at whether it is possible to express this protein in other systems (Mori et al., 2005). Previous studies have shown that is possible to express this protein in various organisms, such as *E. coli* (Giomarelli et al., 2006), tobacco (*N. benthamiana*) (O'Keefe et al., 2009; Hahn et al., 2014; Fuqua et al., 2015a), and transgenic rice endosperm (Vamvaka et al., 2016). Plants have proven to be particularly advantageous for expressing GRFT because of high yields of properly folded, active protein. In addition, plants are typically regarded as a safe production platform for biopharmaceuticals (Hoelscher et al., 2018). Therefore, current research has focused on how to separate manufacturing and processing from plant growth and how to optimize outdoor plant-based pharmaceutical manufacturing methods. Many studies have focused on how proteins of interest may be stored without loss of activity for both short- and long-term use (Fuqua et al., 2015b; Vamvaka et al., 2016; Hoelscher et al., 2018).

Silaging provides an efficient method of storing plant material for long-term periods by preserving green or wet crops under anaerobic conditions allowing fermentation. Silage is typically used for animal feed purposes and allows livestock to be fed in winter when other food sources may not be available. Silage is made by harvesting crops, shredding the plant material, and packing the material tightly to purge as much oxygen as possible. Silage is typically stored in a silo, which is a typically oxygen limiting container that does not allow oxygen to come into contact with the plant material. This allows the silage to undergo anaerobic fermentation and produces acid to create a hostile environment for micro-organisms that may cause spoilage (Driehuis et al., 2018). While ensiled plant material is common for livestock, ensiling plant material for plant-based pharmaceuticals (pharming) was proposed by Hahn et al. (2014).

In the following study we explored the feasibility and practicality of silage as a method of storing GRFT expressed in *N. excelsiana*. Using silage as a storage method for antiviral proteins would allow large-scale outdoor growth, a minimally environmentally controlled storage option for shipment of proteins, separates upstream and downstream phases of manufacturing, and reduces the industry need for multiple growth areas to maintain a continuous supply of biomass.

## Methods

### Plants

*N. excelsiana* is an interspecific hybrid derived from a cross between *N. excelsior* and *N. benthamiana*. Seed was kindly provided by Kentucky Bioprocessing (Pogue et al., 2010, 2014; Shamloul et al., 2014).

### Expression and Plant Growth

*N. excelsiana* plants were infected with a TMV-based virion carrying the RNA expression cassette for GRFT, similar to previous techniques used to express GRFT in *N. benthamiana* (O'Keefe et al., 2009). Briefly, plants were grown in controlled indoor environment with a 16-h light and 8-h dark cycle in 8 separate batches. Batches of *N. excelsiana* were sown over a six-week period and inoculated with either 50 or 150 μg per plant of GRFT-expressing virion from 20 to 31 days after first sown. All plant material above the soil level was harvested 14 days after inoculation and rough chopped. A portion of the rough chopped plant material was used for expression characterization and the remaining material was ensiled.

### Ensiling, Purification, and Assessment

Ensiling was accomplished by rough chopping the plant material, wilting or drying, and then packing into silos. Plant material was compressed with a pneumatic press to remove most of the air and then sealed to begin the anaerobic fermentation process. The plant material packed silos were then stored at ambient temperature and humidity for the duration of the study, 116–146 days. After a fixed storage duration was completed, the plant material was unpacked and homogenized in a blender with sodium acetate buffer at pH 4. GRFT was extracted using this pH 4 buffer, heating to 55°C, and incubating overnight with a bentonite MgCl_2_ mixture (Fuqua et al., 2015b). GRFT activity was assessed using gp120 ELISA and SDS-PAGE tests.

### SDS-PAGE and Densitometry

SDS-PAGE was used to separate and analyze the proteins after extraction. To 45 μL of each sample, 15 μL of 4X SDS loading dye with 2-mercaptoethanol was added. A gel was loaded with the samples and a ladder in 1X XT MES buffer. Electrophoresis was at 200 V for 45 min. Coomasie stain was poured onto the gel, microwaved for 30 s, and rotated at 60 rpm for 25–30 min. The gel was then rinsed in DI water, and the destain was added to the gel. The gel was microwaved for 30 s and rotated at 60 rpm for 30 more minutes. Gels were imaged on a Kodak Image station 4000R Pro and densitometry analysis was done using Carestream SE M software to analyze amount and size of the proteins found in the samples. A standard curve was generated using known concentrations of GRFT (1, 3, 5, 10 μg) vs. their gel intensity. Unknown samples were then fit to the curve and concentrations were calculated.

### GP120 ELISA

GP120-binding ELISAs were used to assess glycan-binding activity of GRFT. A 96-well plate was coated with 0.1 ml gp120 at 0.25 μg/mL overnight at 4°C. The plate was then blocked for a minimum of 2 h with a 3% BSA solution in 1X PBS with 0.05% Tween-20 (PBS-T) using 0.3 mL per well. The plate was washed with PBS-T, and GRFT was added to the wells. The GRFT had a starting concentration of 250 ng/mL and a 2X dilution in 1X PBS was done across the plate. The plate was incubated for 1 h and then washed. The primary antibody was a rabbit anti-GRFT polyclonal serum diluted 1:10,000 in 1X PBS, 0.1 mL per well for 1 h, and then the plate was washed. The secondary antibody was HRP-conjugated goat anti-rabbit diluted to 1:25,000 in 1X PBS, 0.1 mL per well for 1 h, and then the plate was washed. 0.1 mL of TMB solution was added to the wells, developed for 8 min, and then 1M H_2_SO_4_ was added to stop the reaction. The plate was read at 450 nm using a plate reader and plotted optical density (OD_450_) vs. Log of concentration and a four parameter non-linear regression was used to fit a curve and extrapolate the EC_50_.

### Statistical Analysis

All statistical analysis was performed using Graph Pad PRISM® 6.0.

## Results

### Expression in *N. excelsiana*

On eight separate occasions *N. excelsiana* plants were inoculated with griffithsin virion through a high-pressure spray and allowed to grow for 14 days. *N. excelsiana* plants averaged a per plant biomass yield of 86.15 ± 5.05 g. Plant extract samples showed only two bands on SDS-PAGE, at approximately 13 and 19 kDa. The molecular weights correspond to GRFT and TMV coat protein, respectively, as previously reported with expression in *N. benthamiana* (O'Keefe et al., 2009; Fuqua et al., 2015b). Each of the eight batches were quantified for protein yield and griffithsin expression was determined by SDS-PAGE and densitometry (Table 1). The average expression was 344 ± 129 mg/kg and ranged from 214 to 584 mg/kg. We observed that inoculation of plants less than 26 days post-sow (dps) provides a trend (*p*-value of 0.09) toward higher yields (404 ± 155 mg/kg) vs. plants inoculated at or beyond 31 dps (244 ± 38 mg/kg), when analyzed by a two tailed Student's *t*-test. After quantification of GRFT expression levels a portion of the plant material from each was then directed to the ensiling process.

**Table 1:** *N. excelsiana* growth and inoculation parameters.

	**H1**	**H2**	**H3**	**H4**	**H5**	**H6**	**H7**	**H8**
Plant Number	12	6	12	24	24	12	8	24
Plant Biomass (kg)	0.981	0.492	0.952	2.241	2.130	1.104	0.680	2.085
Inoculation time (days post-sow)	26	20	26	31	31	26	26	31
Harvest time (days post-inoculation)	14	14	14	14	14	14	14	14
Initial GRFT Yield (mg/kg of wet mass)	466	584	360	232	287	371	239	214
Silaged				•	•	•	•	•

*Records from eight N. excelsiana growth, inoculation, and harvest cycles and the resulting protein yields*.

### Ensiling

**Figure 1:**
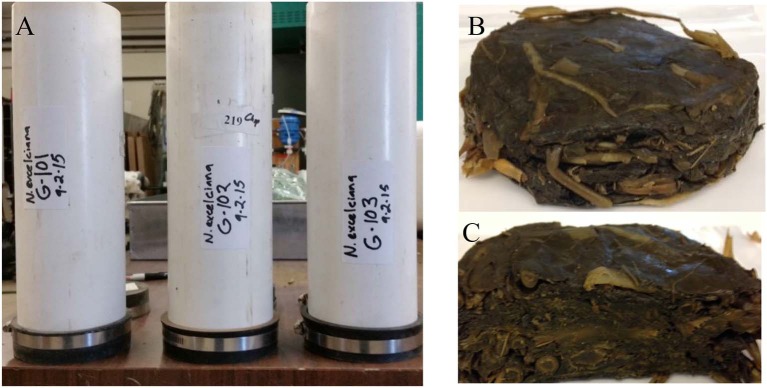
Example of Silo construction and silaged plant material after 16 weeks. **(A)** Silos were constructed out of 4 inch PVC, two air tight caps, and a one-way valve to vent gas during the silage process. **(B)** Approximately 500 g of plant material was compressed within the silos and the anaerobic fermentation process converts sugars to acids and acidifies the tissue. The resulting ~160 g plug of silage plant material is removed from the silo and **(C)** was cut in half, so the layers of material could be viewed.

The first three harvests were not ensiled, but were used to evaluate product expression before committing to the ensiling process. Once expression in *N. excelsiana* was established we ensiled the next five harvests in nine different silos after GRFT expression was quantified. The silos consist of sealed pvc pipes with a one-way valve to release gases from the fermentation process (See Figure 1A). The average amount of plant material that was successfully ensiled was 0.56 kg (Range: 0.372–1.0 kg). Plant material was allowed to wilt after harvest because of concerns that water content in *N. excelsiana* was too high to allow successful silage (Queiroz et al., 2018). Ensiled material was removed from the silos and the plug or puck -like plant material was homogenized with buffer in a blender (See Figures 1B,C).

### Recovery and Activity of Ensiled GRFT

**Figure 2:**
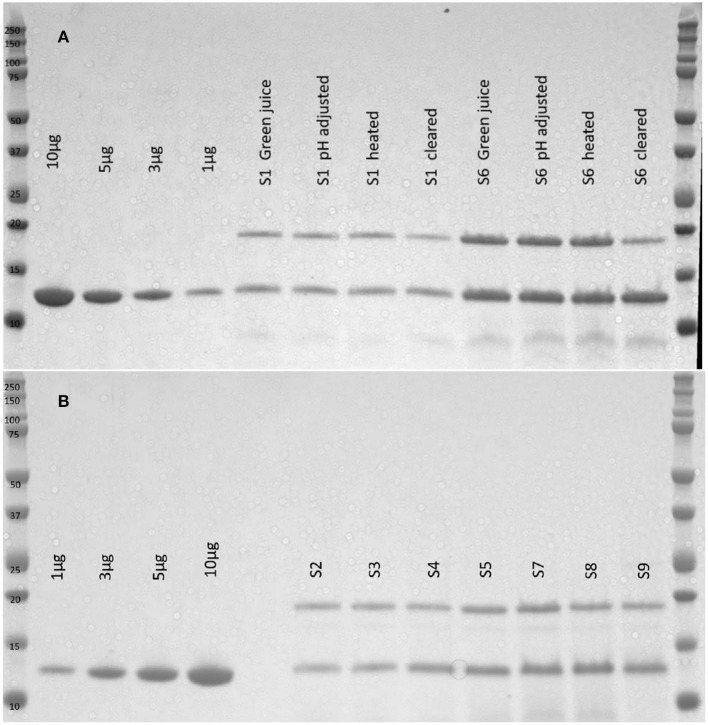
SDS-PAGE of silage extracts. Plant material from silos 1 and 6 was homogenized with 100 mM Sodium Acetate pH4 buffer, adjusted to pH4, heated to 55°C for 15 min, and centrifuged. The process samples and resulting clarified extracts were visualized with SDS-PAGE **(A)**. There are few protein impurities and minimum GRFT loss as the material is processed. Four concentrations of purified GRFT are used as a standard and the primary impurity is TMV coat protein. Silos 2–5 and 7–9 were unpacked and materials were homogenized with sodium acetate buffer and the resulting homogenate was visualized with SDS-PAGE **(B)**. Four concentrations of purified GRFT are used as a standard and the extract yields two primary bands, a ~19 kDa TMV coat protein band and a ~13 kDa GRFT band.

The tissue homogenate (green juice) was evaluated by SDS-PAGE. GRFT yields were quantified from densitometry (Figure 2). A subset of silage tissues was clarified with heat and bentonite as previously described in Fuqua et al. (2015b) to evaluate the implications of silage material on the purification process. Ensiling plant material causes a reduction in plant mass over time with an average of 59% reduction in biomass. The average percent recovery of GRFT from all silos was calculated based upon the tissue mass and expression level of the plant material at the time of ensiling vs. the total GRFT recovered from the ensiled material. We recovered approximately 62.8% (Range: 28.7–97.1%) of the GRFT with 161 mg/kg after silage (Range: 66.7–230.5 mg/kg) (See Table 2).

**Table 2:** Silage parameters.

	**S1**	**S2**	**S3**	**S4**	**S5**	**S6**	**S7**	**S8**	**S9**
Initial GRFT Yield (mg/kg of wet mass)	232	232	232	287	287	371	239	214	214
Silage Duration (Days)	133	146	146	138	138	124	130	117	116
Mass in Silo (kg)	0.424	0.424	0.372	0.555	0.536	0.670	0.497	1.00	0.585
Mass out of Silo (kg)	0.163	0.187	0.171	0.315	0.231	0.187	0.131	0.608	0.165
% Mass loss	61	56	54	43	57	72	74	39	72
Total GRFT in Silage (mg)	44.0	28.3	36.1	127.9	98.7	154.8	65.5	198.4	121.6
Silage GRFT Yield (mg/kg of wet mass)	103.8	66.7	97.0	230.5	184.1	231.1	131.7	198.4	207.9
% Recovery	44.7	28.7	41.7	80.4	64.2	62.4	55.0	92.7	97.1

*Listing of the nine silos filled with griffithsin expressing N. excelsiana and parameters from the resulting silage material*.

**Figure 3:**
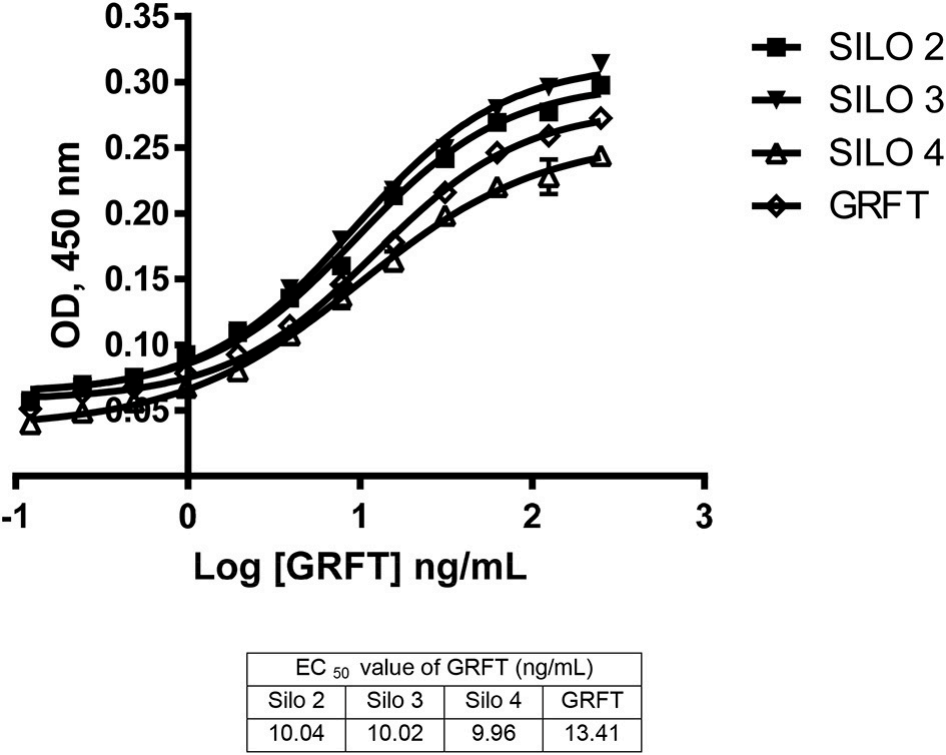
Activity of silage extracts in a gp120 ELISA. Activity from silos 2–4 was assessed through a gp120 ELISA and all show a similar EC_50_ to the standard purified GRFT product.

Activity levels of GRFT were tested after silage against purified GRFT control samples and showed comparable activity. GRFT from silos 2–4 were tested in a gp120 ELISA and compared to a purified control product (Figure 3). Silos 2–4 were chosen because they represent two different *N. excelsiana* harvests and were ensiled for the longest period of any of our silos, 138 or 146 days. The recovered silage products had an average EC_50_ value of 10.01 ± 0.04 ng/mL vs. the control levels of 13.41 ± 2.08 ng/mL.

## Discussion

GRFT has been shown to be a promising new topical microbicide that can neutralize many viruses with extremely small concentrations of the compound by binding to terminal mannose units on oligosaccharides that are present on the surface of viral envelopes. In addition, GRFT has been shown to be safe and effective in various conditions, both *in vivo* and *in vitro*. Previous research has shown GRFT can be expressed in plants, rice, and even in *E. coli*. Additional studies have demonstrated that GRFT can be stored in rice or in dried plant material for extended periods of time. This study provides evidence that it is possible to scale up current methods of viral-vector based GRFT manufacturing by expression in *N. exclesiana*, a cultivar that can be grown outdoors, unlike the conventional production host *N. benthamiana* (Pogue et al., 2010, 2014). We were able to express GRFT in *N. excelsiana* with an average of yield of 344 ± 129 mg/kg, which is approximately half of what is normally expressed in *N. benthamiana* plants. However, the biomass per plant is nearly double an average *N. benthamiana* plant and therefore, yields nearly the same amount of protein per plant (Shamloul et al., 2014). The expression values and optimization related to the timing of infection were performed in a controlled indoor growth environment, which allows for evaluation relative to past *N. benthamiana* expression.

*N. excelsiana* has the advantage of being developed for outdoor plant growth and should be able to be used in a more agricultural scale, allowing field production of GRFT (Pogue et al., 2010, 2014). In previous technoeconomic analysis GRFT production utilities accounted for over 34% of the total cost of goods (Alam et al., 2018). Growing outdoors would eliminate some of those costs and simultaneously reduce the capital investment needed to build a plant growth facility. Additionally, outdoor production of GRFT expressing plants may allow local biopharmaceutical manufacturing in areas with the greatest product needs. GRFT is being developed as an HIV prophylactic and currently the largest market would be in sub-Saharan Africa. For this indication using existing local infrastructure to express the protein may provide the best mechanism to meet potential product demands (Fischer et al., 2013).

We believe that manufacturing GRFT products using outdoor pharming will have overall lower start-up costs than the same product expressed in plants in indoor controlled growth environments. However, advantages of outdoor pharming may be offset by additional burdens caused by environmental related controls. Environmental monitoring of expression vector release will be required and there is the potential for enhanced material processing to remove environmental contaminants. We are proposing to begin piloting outdoor growth experiments to investigate yield and expression parameters that would provide data for revising our technoeconomic model to include outdoor pharming as our source of plant material. The technoeconomic analysis will account for cost advantages of outdoor pharming, as well as, for additional expenditures and provide an unbiased cost-estimate of GRFT production.

Another important potential issue to understand is that to perform year-round manufacturing with an outdoor crop you would need to grow in a climate conducive to continuous growth or identify a cost-effective stable storage solution for your plant material. Even in a climate conducive to year-round plant growth, manufacturing yields would be subject to environmental fluctuations. By separating production and processing, several growing areas can be potentially planned out, and harvests can be shipped to a central processing or holding facility where they await purification into a final pharmaceutical product. Silaging GRFT has been discussed and the concept was piloted with leaf tissue vacuum-sealing (Hahn et al., 2014). However, the bench scale experiment performed by Hahn et al. (2014) does not meet the requirement for a storage method for bulk biomass that would feed a production facility. In this report we discuss methodologies for storing kilogram quantities of GRFT-expressing biomass in a manner that requires minimal environmental controls and little biomass manipulation. The practice was modeled after current agricultural practices and could be scaled to accommodate field production. GRFT was recovered from silage stored at ambient temperatures for > 100 days with no observed impact on potency and product recoveries ranging from 28.7 to 97.1%. The high degree of variation in recovery needs to be explored in depth to identify the critical parameter and make the ensiling process more consistent. We hypothesize the most critical aspect to successful and consist ensiling is managing the liquid content in the plant material. The liquid content of the ensiled material impacts the success of the fermentation process and lost liquid during compression of the plant material likely includes some of the protein of interest. Drying or wilting the plant material in the field before ensiling may improve the yields by limiting the amount of expressed protein that escapes the silo. Additionally, the ensiling process reduces the biomass of the plant material, which increases the concentration of GRFT. The reduced processing burden have favorable cost and process implications that should be explored by alternative technoeconomic studies (Tuse et al., 2014).

The acidification process imparted by ensiling tissue is not likely conducive to the stability of all proteins and seems to degrade the majority of plant proteins present in the tissue. However, GRFT and TMV coat protein were both stable through the process, but they both have pIs below 5.5. Interestingly, TMV has been explored as an antigen carrier in many subunit vaccines and may be another target for silage storage (Mallajosyula et al., 2014; Banik et al., 2015; McCormick et al., 2018). In the future we will evaluate the infectivity of TMV and the stability of the virion to better understand if it would be suitable for antigen presentation.

This study confirms previous reports on the viability of *N. excelsiana* as a plant expression host (Pogue et al., 2010; Shamloul et al., 2014) and is the first report of GRFT being expressed in *N. excelsiana*. The data reported here necessitates evaluation in the context of outdoor pharming with a technoeconomic analyses as the primary metric to determine viability. The data support silage as a viable and scalable method to store GRFT for long-term needs. The recovery led to a high yield of GRFT, and methods for storing the silage can be optimized using our study findings. Utilizing silage allows for ease of transportation and storage, as silage is extremely stable and requires relatively small amounts of space relative to the yield. The study may be applied in whole with *N. excelsiana* expression supported by ensiling techniques or may be more useful independently, with the ensile process used with *N. benthamiana*. Our preliminary evaluation of ensiling *N. benthamiana* has resulted in similar positive outcomes. In conclusion, this study showed that growing GRFT expressed in *N. excelsiana* at an agricultural scale and ensiled for storage is a method that needs to be explored for cost and simplicity advantages to help understand the processes potential.

## Data Availability Statement

The datasets generated for this study are available on request to the corresponding author.

## Author Contributions

JC performed the griffithsin analytical experiments and data analysis. RM ensiled the plant material and provided guidance on the scalability of silage. PE drafted the manuscript. KP provided scientific guidance on griffithsin and plant viral vectors. JF directed the experiments, performed data analysis, and drafted parts of the manuscript. All authors reviewed and provided corrections to the manuscript.

### Conflict of Interest

KP and JF are founders of GROW Biomedicine, LLC that is exploring commercialization of griffithsin technologies. The remaining authors declare that the research was conducted in the absence of any commercial or financial relationships that could be construed as a potential conflict of interest.
